# Rufinamide, a Triazole-Derived Antiepileptic Drug, Stimulates Ca^2+^-Activated K^+^ Currents While Inhibiting Voltage-Gated Na^+^ Currents

**DOI:** 10.3390/ijms232213677

**Published:** 2022-11-08

**Authors:** Ming-Chi Lai, Sheng-Nan Wu, Chin-Wei Huang

**Affiliations:** 1 Department of Pediatrics, Chi-Mei Medical Center, Tainan 71004, Taiwan; 2 Department of Physiology, College of Medicine, National Cheng Kung University, Tainan 70101, Taiwan; 3 Institute of Basic Medical Sciences, College of Medicine, National Cheng Kung University, Tainan 70101, Taiwan; 4 Department of Neurology, National Cheng Kung University Hospital, College of Medicine, National Cheng Kung University, Tainan 70101, Taiwan

**Keywords:** rufinamide (1-((2,6-difluorophenyl)methyl)-1H-1,2,3-triazole-4carboxamide), antiepileptic drug, Ca^2+^-activated K^+^ current, large-conductance Ca^2+^-activated K^+^ channel, hysteresis, voltage-gated Na^+^ current

## Abstract

Rufinamide (RFM) is a clinically utilized antiepileptic drug that, as a triazole derivative, has a unique structure. The extent to which this drug affects membrane ionic currents remains incompletely understood. With the aid of patch clamp technology, we investigated the effects of RFM on the amplitude, gating, and hysteresis of ionic currents from pituitary GH_3_ lactotrophs. RFM increased the amplitude of Ca^2+^-activated K^+^ currents (*I*_K(Ca)_) in pituitary GH_3_ lactotrophs, and the increase was attenuated by the further addition of iberiotoxin or paxilline. The addition of RFM to the cytosolic surface of the detached patch of membrane resulted in the enhanced activity of large-conductance Ca^2+^-activated K^+^ channels (BK_Ca_ channels), and paxilline reversed this activity. RFM increased the strength of the hysteresis exhibited by the BK_Ca_ channels and induced by an inverted isosceles-triangular ramp pulse. The peak and late voltage-gated Na^+^ current (*I*_Na_) evoked by rapid step depolarizations were differentially suppressed by RFM. The molecular docking approach suggested that RFM bound to the intracellular domain of K_Ca_1.1 channels with amino acid residues, thereby functionally affecting BK_Ca_ channels’ activity. This study is the first to present evidence that, in addition to inhibiting the *I*_Na_, RFM effectively modifies the *I*_K(Ca)_, which suggests that it has an impact on neuronal function and excitability.

## 1. Introduction

Rufinamide (RFM, Banzel^®^, Inovelon^®^, 1-((2,6-difluorophenyl)methyl)-1H-1,2,3-triazole-4carboxamide), is recognized as a unique anticonvulsant drug because, as a triazole derivative, its structure is dissimilar to other currently marketed antiepileptic drugs [1,2,3,4]. It is increasingly being used in combination with other medications and therapies to treat Lennox-Gastaut syndrome, severe epileptic encephalopathy, and other seizure disorders [3,4,5,6,7,8,9,10,11,12,13,14,15,16,17,18,19,20,21,22,23,24,25,26]. RFM has been shown to decrease the incidence and severity of seizures associated with Lennon-Gastaut syndrome with a favorable cognitive side-effect profile [6,21,22,27]. However, the answers that have been provided so far to the questions of whether and how RFM causes any integrated effects of transmembrane ionic currents are rather speculative.

Although its mechanism of action as an antiepileptic drug is still unclear, RFM modulates the activity of Na_V_ channels by prolonging the inactive state of these channels [28,29,30,31,32,33,34] and the specific stabilization of the intermediate inactivated Na_V_ channels [35]. It has previously been shown to improve functional and behavioral deficits through a mechanism linked to its blockade of tetrodotoxin-resistant Na^+^ currents [32]. However, whether this drug modifies the amplitude and gating of other types of ionic currents is unclear.

RFM has been shown to exert a neuroprotective effect against kainic acid-induced excitotoxic neuronal death in the hippocampus [36,37]. It has also shown promise by improving cognition and ischemia-reperfusion injury and by increasing neurogenesis in the gerbil hippocampus [38]. It has also proven beneficial in reducing myotonia in skeletal muscles [39]. However, there is evidence that atonic seizures may be aggravated by RFM [40].

Large-conductance Ca^2+^-activated K^+^ (BK_Ca_ or BK) channels (KCa1.1, KCNMA1, *Slo1*) belong to a family of voltage-gated K^+^ channels, and they are activated by an increase in either the cytosolic concentration of Ca^2+^ or the membrane potential, or both. The activation of BK_Ca_ channels can conduct large amounts of K^+^ ions across the cell membrane. Owing to its high-conductance state and a single-channel conductance of about 150–250 pS, the BK_Ca_ channel is also thought to be a maxi- or large-K^+^ channel. This family of K^+^ channels is functionally expressed in an array of excitable and non-excitable cells, and its activity can play a role in numerous physiological or pathological events, including membrane excitability, neurotransmitter release, stimulus-secretion coupling, muscle relaxation, and motor coordination [41,42,43,44,45,46,47,48]. Small natural and synthetic molecules have been shown to be important regulators of BK_Ca_-channel activity [45,49,50,51,52]. It has also been noted that the BK_Ca_ channel shows promise in approaches to treating epilepsy [41,42,43,53,54].

In view of the considerations stated above, the purpose of this work is to establish whether RFM causes any modifications in membrane ionic currents in electrically excitable cells (e.g., pituitary GH_3_ lactotrophs). The types of membrane ionic currents that we studied include the Ca^2+^-activated K^+^ current (*I*_K(Ca)_) and the voltage-gated Na^+^ current (*I*_Na_). The present study is the first to present evidence that, in addition to inhibiting the *I*_Na_, RFM is capable of effectively stimulating activity in BK_Ca_ channels.

## 2. Results

### 2.1. Effect of RFM on CA^2+^-Activated K^+^ Current (I_K(CA)_) Identified in GH_3_ Cells

**Figure 1:**
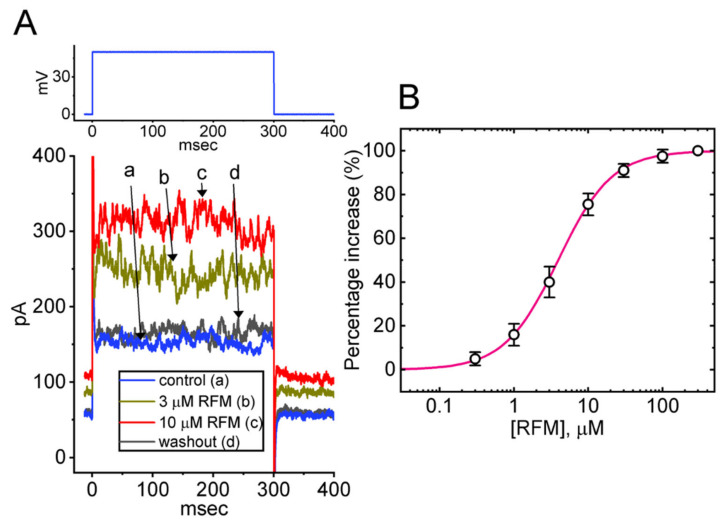
Effect of RFM on Ca^2+^-activated K^+^ current (*I*_K(Ca)_) measured from pituitary GH_3_ cells. Cells were bathed in normal Tyrode’s solution containing 1.8 mM CaCl_2_, the ionic composition of which is detailed in Materials and Methods. (**A**) Representative *I*_K(Ca)_ traces obtained in the control (i.e., absence of RFM, a), during the exposure to 3 μM RFM (b) or 10 μM RFM (c), and after washout of RFM (d). The voltage-clamp protocol is illustrated in the upper part. (**B**) Concentration-dependent stimulation of RFM on *I*_K(Ca)_ in response to membrane depolarization (mean ± SEM; *n* = 8 for each point). Current amplitude was measured at the end of the 300 ms depolarizing step to +50 mV from a holding potential of 0 mV. The *I*_K(Ca)_ amplitude during cell exposure to 300 μM RFM was taken as 100%, and those in the presence of different RFM concentrations were thereafter compared. The Hill equation indicated in Materials and Methods was well-fitted to the experimental data (solid red line).

In the first stage of experiments, the modification of the *I*_K(Ca)_ caused by exposing GH_3_ cells to RFM was evaluated. We bathed the cells in normal Tyrode’s solution, which contained 1.8 mM CaCl_2_, and the recording pipette was backfilled with a K^+^-containing isotonic solution. The membrane patch under the electrode tip was broken through by gentle suction in order to conduct the whole-cell current recordings. We applied the voltage clamp technique to each tested cell at 0 mV, and we subjected it to a 300-ms depolarizing voltage command pulse to +50 mV. Under this experimental protocol, a large and noisy outward current in response to the depolarizing step was robustly evoked, and the magnitude of the current evidently rose with increasing membrane depolarizations. Such ionic currents have been characterized as *I*_K(Ca)_ [52,53,54,55]. Of note is the finding that, after the cells had been exposed for 1 min to RFM at a concentration of 3 μM or 10 μM, a concentration-dependent increase in the amplitude of the *I*_K(Ca)_ was observed (see Figure 1A). More specifically, compared to a control value of 153 ± 19 pA (*n* = 8), the addition of 3 μM RFM produced an increase in the current amplitude at the end of the step depolarization to 243 ± 32 pA (*n* = 8, *p* < 0.05), whereas the addition of 10 μM RFM produced an increase to 298 ± 39 pA (*n* = 8, *p* < 0.05). After washing out the RFM, the current amplitude returned to 159 ± 21 pA (*n* = 8).

The varying stimulating effects of different concentrations of RFM on the amplitude of the *I*_K(Ca)_ elicited by the 300 ms depolarizing step were further examined, with the effects on GH_3_ cells shown in Figure 1B. According to a Hill equation detailed in the section titled Materials and Methods, the EC_50_ value of RFM required for evoking the *I*_K(Ca)_ was estimated to be 3.9 μM, with a Hill coefficient of 1.2.

### 2.2. Effect of RFM on the Current-Voltage (I–V) Relationship the I_K(Ca)_ in GH_3_ Cells

**Figure 2:**
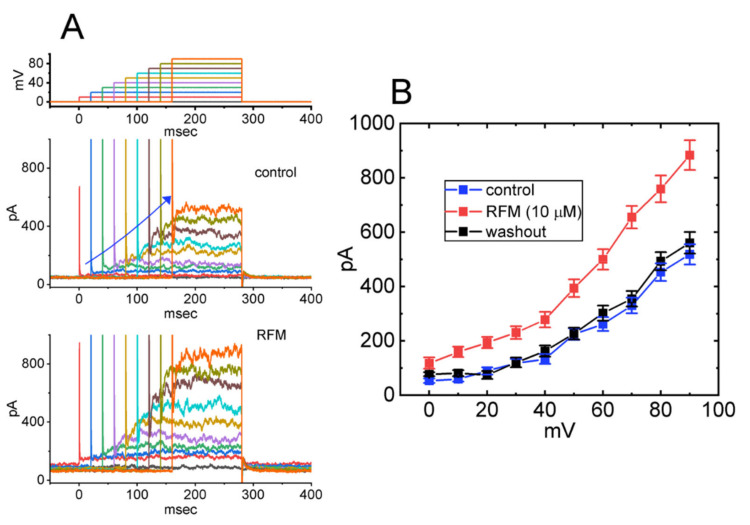
Effect of RFM on mean current-voltage (*I–V*) relationships of *I*_K(Ca)_ identified in GH_3_ cells. (**A**) Representative current traces obtained in the absence (upper) and presence (lower) of 10 μM RFM. The uppermost part shows the voltage-clamp protocol applied. The potential traces labeled in different colors correspond with current ones acquired without or with the RFM application. The duration in each depolarizing step is different for better illustrations, and the blue solid arrow indicates the outwardly rectifying properties of *I*_K(Ca)_ with increasing positive voltage. (**B**) Mean *I–V* relationships of *I*_K(Ca)_ amplitude acquired in the control (blue squares), during exposure to 10 μM RFM (red squares) and washout of RFM (black squares) (mean ± SEM; *n* = 8 for each point). Current amplitude was measured at the end of each depolarizing step.

We next investigated the effect of RFM on the *I*_K(Ca)_ for various membrane potentials. In these experiments, the *I*_K(Ca)_ was robustly evoked as each tested cell was depolarized from 0 mV by a series of depolarizing voltage steps to a range of potentials between 0 mV and +90 mV in 10-mV increments (see Figure 2A). The mean *I–V* relationships of the *I*_K(Ca)_ with and without the addition of RFM are shown in Figure 2B. The whole-cell conductance of the *I*_K(Ca)_ was measured at membrane potentials between +40 mV and +90 mV, and the addition of 10 μM RFM caused the *I*_K(Ca)_ conductance to increase from 7.2 ± 0.6 nS to 15.1 ± 0.9 nS (*n* = 8, *p* < 0.05). After washing out the RFM, the conductance returned to 7.3 ± 0.7 nS (*n* = 8).

### 2.3. Comparisons among the Effects of RFM Only, RFM Plus Apamin, RFM Plus Glibenclamide, RFM Plus Iberiotoxin, and RFM Plus Paxilline on the Amplitude of the I_K(Ca)_

**Figure 3:**
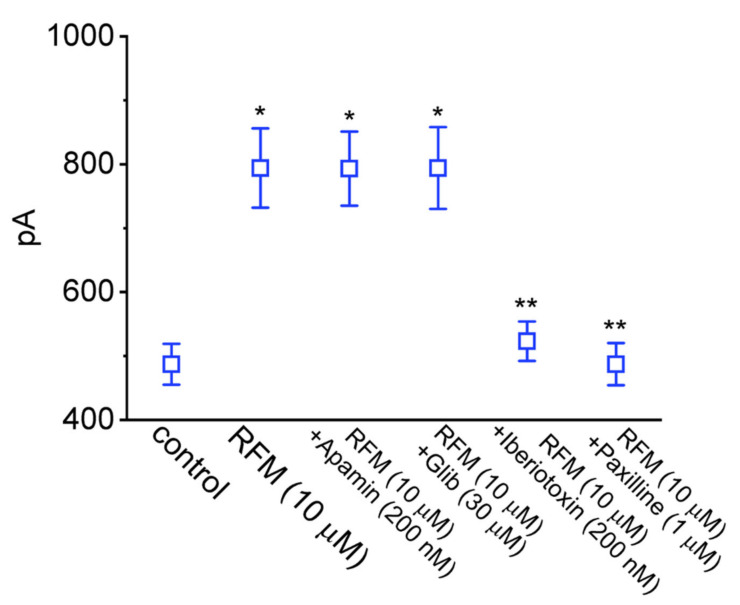
Summary scatter graph showing comparisons on *I*_K(Ca)_ amplitude produced in the presence of RFM, RFM plus apamin, RFM plus glibenclamide (Glib), RFM plus iberiotoxin, and RFM plus paxilline in GH_3_ cells. In these experiments, *I*_K(Ca)_ was evoked by a 300 ms depolarizing pulse from 0 to +50 mV, and the current amplitude was measured at the end of the command pulse. Each point represents the mean ± SEM (*n* = 8). * Significantly different from control (*p* < 0.05) and ** significantly different from RFM (10 μM) alone group (*p* < 0.05).

We next examined whether the subsequent addition of apamin, glibenclamide, iberiotoxin, and paxilline, while retaining the RFM, would alter the stimulatory effect of RFM on the *I*_K(Ca)_ in GH_3_ cells. Evidence has been found that apamin blocks the activity of small-conductance Ca^2+^-activated K^+^ channels, glibenclamide is an inhibitor of ATP-sensitive K^+^ (K_ATP_) channels, and iberiotoxin and paxilline are effective at suppressing BK_Ca_-channel activity [51,56,57]. As demonstrated in the scatter graph presented in Figure 3, in the continued presence of 10 μM RFM, the addition of neither apamin nor glibenclamide modified the RFM-induced increase in the amplitude of the *I*_K(Ca)_. However, the subsequent addition of iberiotoxin and paxilline reversed the stimulation of the *I*_K(Ca)_ produced by the presence of RFM. It, thus, seems likely that the RFM-mediated stimulation of the *I*_K(Ca)_ is caused predominantly by the current flow through the BK_Ca_ channels present in GH_3_ cells.

### 2.4. Stimulatory Effect of RFM on Large-Conductance CA^2+^-Activated K^+^ (BK_Ca_) Channels in GH_3_ Cells

**Figure 4:**
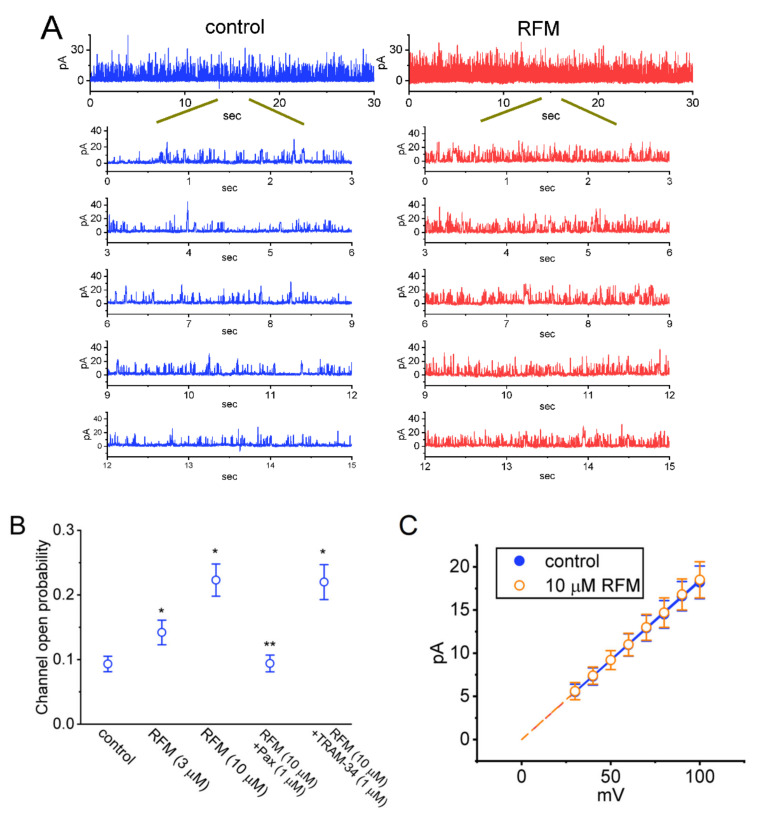
Effect of RFM on the activity of large-conductance Ca^2+^-activated K^+^ (BK_Ca_) channels recorded from GH_3_ cells. The single-channel experiments were conducted as cells were exposed to high-K^+^ concentration (145 mM) containing 0.1 μM Ca^2+^, the pipette solution contained K^+^-enriched solution, and an inside-out configuration with a holding potential of +60 mV was created. (**A**) Representative tracings of BK_Ca_ channels in the absence (left, blue color) and presence (right, red color) of 10 μM RFM. RFM was applied to the bath medium. The lower five traces were expanded records from the uppermost current ones (15 s in duration) in the absence (left) and presence (right) of 10 μM RFM. The upper deflection indicates the opening event of the channel recorded at the holding potential of +60 mV. Of note, the BK_Ca_-channel activity was conceivably enhanced as RFM was applied to the bath (i.e., in the cytosolic leaflet of the detached membrane patch). (**B**) Summary scatter graph demonstrating the effect of RFM, RFM plus paxilline, and RFM plus TRAM-34 on the probability of BK_Ca_-channel openings (mean ± SEM; *n* = 7 for each point). Inside-out current recordings were performed in these experiments, the potential was set at +60 mV, and each tested compound was applied to bath medium. * Significantly different from control (*p* < 0.05) and ** significantly different from RFM (10 μM)-alone group (*p* < 0.05). (**C**) Mean *I–V* relationships of single BK_Ca_-channel currents acquired in the absence (blue filled circles) and presence (orange open circles) of 10 μM RFM (mean ± SEM; *n* = 7 for each point). The dashed linear line was pointed toward 0 mV (i.e., reversal potential). Of note, these two linear *I–V* relationships of BK_Ca_ channels between the absence and presence of RFM are superimposed.

In terms of its biophysical characteristics, the *I*_K(Ca)_ is a large, noisy, voltage-dependent, Ca^2+^-sensitive K^+^ current, and its strength largely comes from the opening of the BK_Ca_ channels inherently in GH_3_ cells [45]. Therefore, we continued to explore the possible effects that RFM has on the activity of BK_Ca_ channels, and to this end, the inside-out patch clamp technique was performed. The recordings of the currents in cells that were bathed in a symmetrical K^+^ concentration (145 mM) and bath medium contained 0.1 μM Ca^2+^. While taking measurements, we kept the examined cell in a voltage clamp at a holding potential of +60 mV. As illustrated in Figure 4A, after the introduction of 10 μM RFM into the part of the ion channel near the cytosolic membrane leaflet, a drastic elevation in the open-state probability of the channel was detected. Recorded from the detached patches of GH_3_ cells, these probabilities for the BK_Ca_ channels significantly and consistently increased from 0.093 ± 0.012 to 0.142 ± 0.019 (*n* = 7, *p* < 0.05) during exposure to 3 μM RFM and to 0223 ± 0.025 (*n* = 7, *p* < 0.05) during exposure to 10 μM RFM. After washing out the compound, the channel activity was reduced to 0.101 ± 0.014 (*n* = 7, *p* < 0.05).

As shown in Figure 4B, in the continued presence of 10 μM RFM, the subsequent addition of 1 μM paxilline effectively attenuated the RFM-stimulated activity of the channels, which was not the case when 1 μM TRAM-34 was introduced. Paxilline is known to be an inhibitor of the BK_Ca_ channels, while TRAM-34 has been shown to suppress the activity of intermediate-conductance Ca^2+^-activated K^+^ (IK_Ca_) channels [57,58]. Moreover, the mean open times of the BK_Ca_ channels in the absence and presence of 10 μM RFM did not differ significantly (2.32 ± 0.11 for the control versus 2.33 ± 0.12 in the presence of RFM, *n* = 7, *p* > 0.05). However, the slow component of the mean closed time of the channels in the presence of 10 μM RFM decreased significantly to 6.17 ± 0.57 (*n* = 7, *p* < 0.05) from a control value of 12.22 ± 0.89 (*n* = 7).

### 2.5. Effect of RFM on the Voltage-Dependent Hysteresis of BK_Ca_-Channel Activity Evoked in Response to a Long-Lasting Inverted Isosceles-Triangular Ramp Pulse

**Figure 5:**
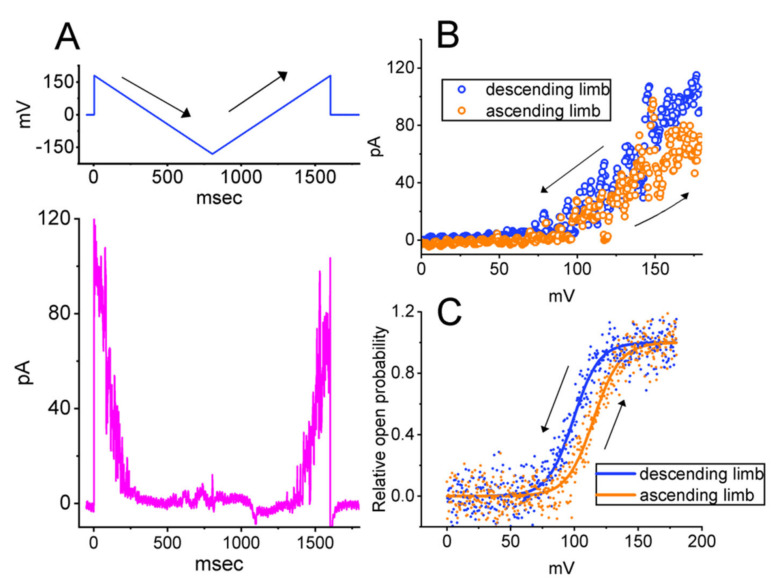

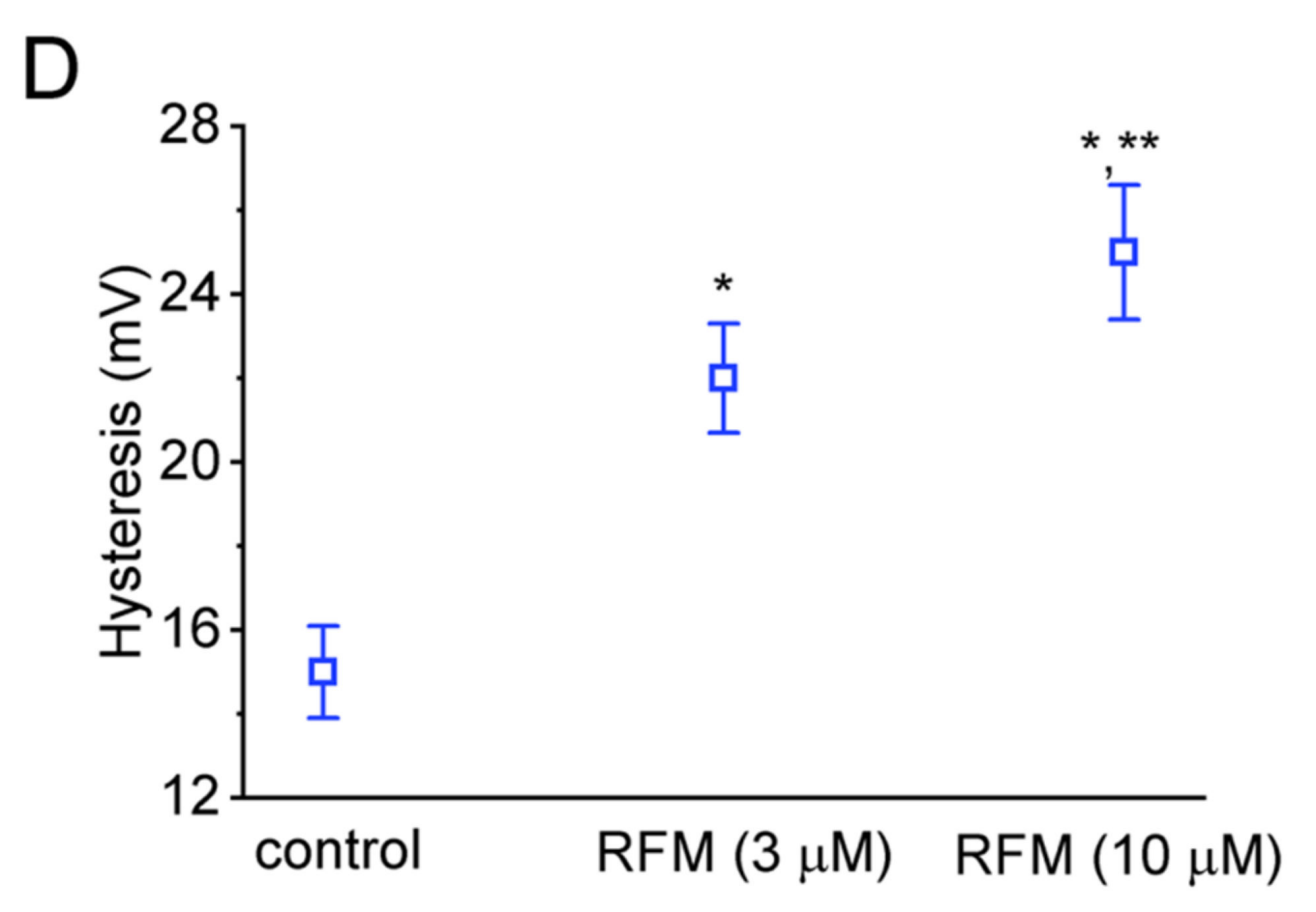
BK_Ca_-channel activity activated by inverted isosceles-triangular ramp pulse obtained with or without the RFM application. Inside-out current recordings were taken, bath medium contained 0.1 μM Ca^2+^, and an inverted triangular ramp pulse between +180 and −180 mV with a duration of 1.6 s (i.e., ramp pulse = ±450 mV/s) was applied to the patch. (**A**) Representative current traces (pink colors) obtained in the control period. The upper part shows the voltage-clamp protocol applied, and the solid arrow indicates the direction in which time passes. (**B**) Instantaneous relationship of BK_Ca_-channel current versus membrane potential evoked by descending (blue open symbols in data points) or ascending limb (organ open symbols) activated during the inverted isosceles-triangular ramp pulse. (**C**) Hysteretic strength of BK_Ca_-channel opening activated by 1.6 s long-lasting isosceles-triangular ramp pulse. Note that the relationship of relative open probability versus membrane potential is overly distinguishable between the descending (forward) and ascending (backward) limbs of the triangular ramp pulse. (**D**) Summary scatter graph showing the effect of RFM (3 or 10 μM) on voltage-dependent hysteresis of BK_Ca_ channels evoked by inverted triangular ramp pulse (mean ± SEM; *n* = 7 for each point). The hysteretic strength was measured at the voltage separation between the descending (forward) and ascending (backward) limb at 50% of the relative open probability. An Inside-out configuration was created, and an inverted isosceles-triangular ramp pulse with a ramp speed of ±450 mV/s was applied to the detached patch. * Significantly different from control (*p* < 0.05) and ** significantly different from RFM (3 μM)-alone group (*p* < 0.05).

The voltage-dependent hysteresis of ionic currents (i.e., a lag in current strength as the linear voltage command is changed in the opposite direction) has recently been shown to have a significant impact reminiscent of electrical activity, as when an action potential fires in a neuron (i.e., initial depolarization and late repolarization) [59,60,61,62]. In view of such findings, we further explored whether voltage-dependent hysteresis occurred in the BK_Ca_-channel activity recorded in GH_3_ cells. In this series of inside-out patch clamp experiments, we used a long (1.6 s in duration) inverted isosceles-triangular ramp pulse with a ramp speed of ±450 mV/s to measure the characteristics of the hysteretic behavior (Figure 5). The trajectory of the channel activity evoked by the downsloping ramp pulse (i.e., a change in voltage from +180 to −180 mV) and the upsloping ramp pulse (i.e., a change from −180 to +180 mV) as a function of time was clearly distinguishable. In other words, in the control phase (i.e., when the RFM was not present), the relative open probability of the channels evoked by the descending (forward) end of the inverted triangular voltage ramp was higher than that in response to the upsloping (backward) end (Figure 5C).

With the goal of quantifying our observations, we evaluated the degree of voltage-dependent hysteresis based on the voltage separation between the downsloping and upsloping branches at 50% of the relative probability of the BK_Ca_ channels being open. In a manner dependent on the concentration used, the presence of RFM increased the overall strength of the hysteresis exhibited by the BK_Ca_ channels in the GH_3_ cells (Figure 5D). More specifically, as the single-channel recordings were taken, the addition of 3 μM RFM led to an increase in the strength of the hysteresis to 22.1 ± 1.3 mV (*n* = 7, *p* < 0.05), whereas the addition of 10 μM RFM increased it to 25.1 ± 1.6 mV (*n* = 7, *p* < 0.05), from a control value of 14.9 ± 1.1 mV (*n* = 7).

### 2.6. Effect of RFM on Voltage-Gated NA^+^ Currents (I_Na_) Recorded in GH3 Cells

**Figure 6:**
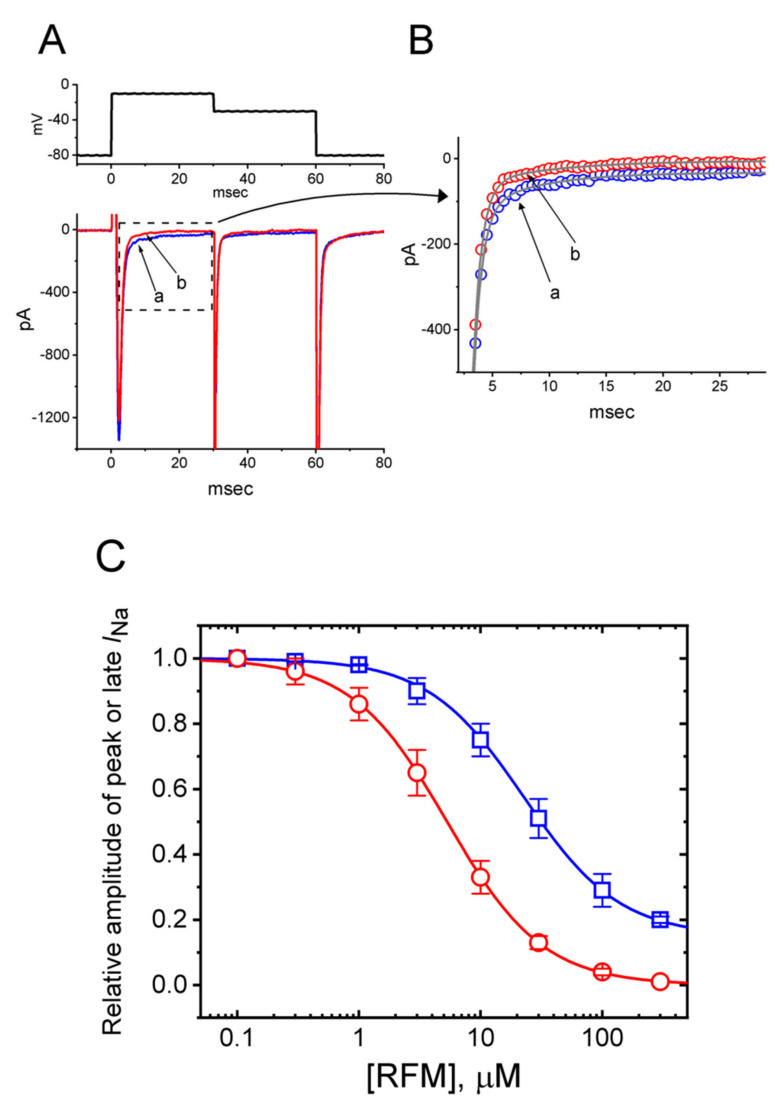
Inhibitory effects of RFM on voltage-gated Na^+^ current (*I*_Na_) identified in GH_3_ cells. The experiments were conducted when cells were bathed in Ca^2+^-free, Tyrode’s solution containing 10 mM TEA and 0.5 mM CdCl_2_, and the electrode that we used was filled with a Cs^+^-enriched solution. (**A**) Representative potential (upper) and current traces (lower) obtained in the control period (a) and during exposure to 3 μM RFM (b). (**B**) Representative expanded traces (indicated on a faster time scale) shown from the dashed box in (**A**). Note that cell exposure to RFM differentially suppresses the peak and late components of *I*_Na_ in response to rapid membrane depolarization. (**C**) Concentration-dependent inhibition of RFM on the peak (blue open squares) and late (red open circles) components of *I*_Na_ (mean ± SEM; *n* = 8 for each point). Current amplitude was taken at the beginning and end of the 30 ms depolarizing step applied from −80 to −10 mV. The modified Hill equation indicated in Materials and Methods was reasonably fitted to the experimental data (blue and red smooth lines).

Earlier studies have demonstrated the effectiveness of RFM in altering the magnitude of the *I*_Na_ in different cell types [28,29,30,31,32,33,34,35]. We further examined whether the addition of RFM would exert any perturbations on the magnitude of the *I*_Na_. In these experiments, to preclude the contamination of Ca^2+^ and K^+^ currents, we bathed the cells in Ca^2+^-free Tyrode’s solution containing 10 mM TEA and 0.5 mM CdCl_2_, and the recording electrodes were backfilled with Cs^+^-containing solution [63,64]. The cells were exposed to 3 μM RFM, and the peak and late components of the *I*_Na_ activated by a 30 ms depolarizing step from −80 mV to −10 mV were robustly decreased. Concomitant with these results, the time course of *I*_Na_ inactivation was shortened in the presence of 3 μM RFM (Figure 6A,B). The application of 3 μM RFM decreased the peak amplitude of the *I*_Na_ to 1201 ± 96 pA (*n* = 8, *p* < 0.05) from a control value of 1287 ± 103 pA (*n* = 8) and the late amplitude of the *I*_Na_ to 21 ± 3 pA (*n* = 8, *p* < 0.05) from a control value of 48 ± 4 pA (*n* = 8). After washing out the RFM, the peak and late *I*_Na_ returned to 1209 ± 99 pA (*n* = 8) and 46 ± 4 pA (*n* = 8), respectively. Furthermore, the exposure of the GH_3_ cells to 3 μM RFM resulted in an escalated time course of *I*_Na_ inactivation, as shown by the reduction in τ_inact(S)_ (the slow component of the inactivation time constant of the *I*_Na_) from 11.2 ± 0.8 ms to 4.1 ± 0.2 ms (*n* = 8, *p* < 0.05).

The effect that the addition of different concentrations of RFM had on suppressing the peak and late *I*_Na_ evoked by the 30 ms depolarizing step was further examined. This inhibitory effect on the amplitude of the *I*_Na_ in the GH_3_ cells is shown in Figure 6C. According to a Hill equation presented in the section titled Materials and Methods, the IC_50_ values of the RFM required for inhibiting the peak and late *I*_Na_ activated by rapid step depolarizations were 22.7 μM and 3.1 μM, respectively.

### 2.7. Effect of RFM on Mean I–V Relationship of the Peak I_Na_ Identified in GH_3_ Cells

**Figure 7:**
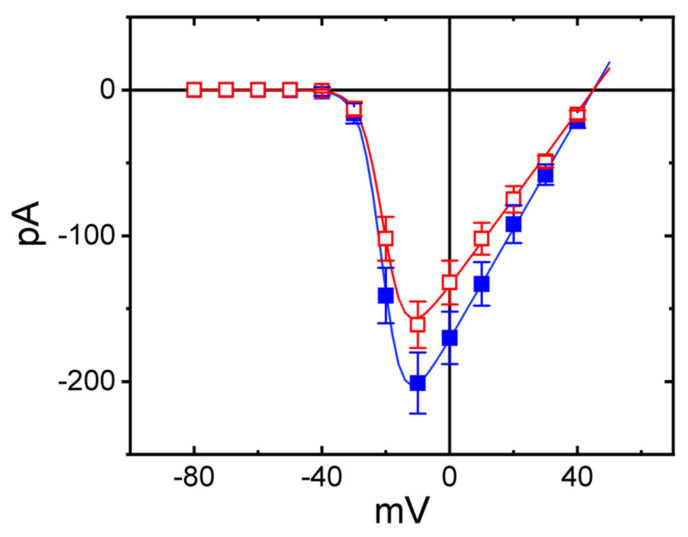
Effect of RFM on mean *I–V* relationship of peak *I*_Na_ in GH_3_ cells. The examined cell was held at −80 mV and a series of voltage steps between −80 and +40 mV in 10-mV increments was thereafter applied to it. Current amplitude measured at the start of the abrupt depolarizing step was taken. The data points indicated in blue-filled squares are controls (i.e., absence of RFM), and those in red open squares were taken in the presence of 3 μM RFM. Each data point represents the mean ± SEM (*n* = 7). The smooth continuous lines were least-squares fitted to a modified Boltzmann function detailed in Materials and Methods.

We further examined the *I–V* relationships of the peak *I*_Na_ obtained with and without the application of RFM. These voltage-clamp experiments were conducted in cells held at −80 mV, and a series of voltage pulses ranging between −80 mV and +40 mV was then applied to the tested cells. As shown in Figure 7, the presence of 3 μM RFM did not alter the overall *I–V* relationship of the peak *I*_Na_, although it did increase the peak amplitude of the *I*_Na_, particularly at the level of −10 mV. The values of the reversal potential and the threshold potential of the peak *I*_Na_ recorded both in the absence and in the presence of RFM were indistinguishable. The *I–V* curves obtained during the control period and during cell exposure to 3 μM RFM were optimally fitted with a Boltzmann function, as indicated in the section titled Materials and Methods (In control: (i.e., the absence of RFM), G = 3.8 *±* 0.3 nS, Vh = −20.8 *±* 1.9 mV, *k* = 3.3 *±* 0.3 (*n* = 8), while in the presence of 3 μM RFM: G = 2.9 *±* 0.3 nS, Vh = −20.4 *±* 1.8 mV, *k* = 3.4 *±* 0.3 (*n* = 8)). It is thus likely that the steady-state activation curve of the *I*_Na_ was not changed during cell exposure to 3 μM RFM, although this concentration of RFM decreased the whole-cell conductance of the peak *I*_Na_.

### 2.8. RFM-Mediated Attenuation of the Stimulation of the I_Na_ Produced by Tefluthrin (Tef)

**Figure 8:**
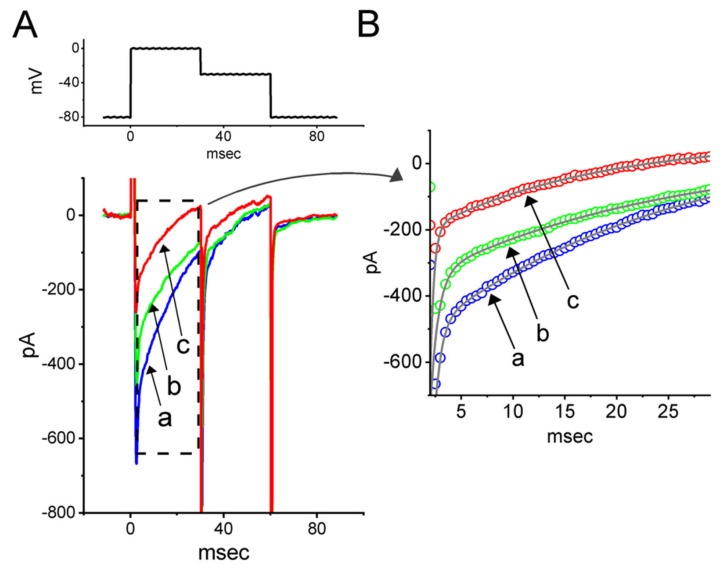
Effect of tefluthrin (Tef) and Tef plus RFM (10 or 30 μM) on *I*_Na_ evoked by the rapid depolarizing step in GH_3_ cells. (**A**) Representative current traces obtained in the presence of 10 μM Tef (a) and during the exposure to 10 μM Tef plus 10 μM RFM (b) or 10 μM Tef plus 30 μM RFM (c). The voltage−clamp protocol applied is displayed in the upper part. (**B**) Current traces taken from the dashed box in (**A**). Of notice, in the continued presence of Tef, further application of RFM remains effective in reversing Tef-stimulated *I*_Na_ in these cells.

Tefluthrin (Tef), a type-I pyrethroid insecticide, has been shown to activate the *I*_Na_ [63,64]. We further investigated whether the subsequent addition of RFM would modify the Tef-activated *I*_Na_ in GH_3_ cells. As shown in Figure 8, when the cells were exposed to 10 mM Tef, the peak amplitude and the τ_inact(S)_ of the *I*_Na_ activated by the application of abrupt step depolarizations from −80 mV to −10 mV were 669 ± 31 pA and 26 ± 9 ms (*n* = 8), respectively. In the continued presence of 10 mM Tef, the further addition of 10 μM RFM reduced the current amplitude and the t_inact(S)_ to 432 ± 27 pA and 21 ± 7 ms (*n* = 8, *p* < 0.05), respectively, whereas adding 30 μM RFM resulted in the current amplitude and the t_inact(S)_ being decreased to 258 ± 19 pA and 16 ± 5 ms (*n* = 8, *p* < 0.05), respectively. It is clear, therefore, that while the cells were exposed to Tef, the addition of RFM was effective at modifying the Tef-activated *I*_Na_ in the GH_3_ cells.

## 3. Discussion

This study has shown that, as cells were continuously exposed to RFM, depending on its concentration, the amplitude of *I*_K(Ca)_ increased with an EC_50_ value of 3.9 μM. The BK_Ca_ channels were stimulated by the presence of RFM, with their activity detected using the inside-out configuration of the patch-clamp technique, although no discernible change was detected in the single-channel conductance. Therefore, the BK_Ca_ channel is expected to be a relevant target for RFM treatment.

In our study, the RFM-mediated increase in BK_Ca_-channel activity in GH_3_ cells was not associated with a change in single-channel amplitude, as was confirmed by the absence of a noticeable difference in the single-channel conductance of the channels measured with and without the addition of RFM. However, the RFM-stimulated activity of the BK_Ca_ channels was attenuated by the further addition of paxilline, which was not the case when TRAM-34 was added. Additionally, the presence of RFM was shown to shorten the slow component of the mean closed time of the channel. This may be a major factor in the RFM-mediated activation of the BK_Ca_ channels, although no change in the mean open time of the channel was found.

It needs to be emphasized that the amplitude of *I*_K(Ca)_ shown in Figure 1A in control conditions at the level of +50 mV was around 150 pA. Since the single-channel conductance of the BK_Ca_ channels was estimated to be around 200 pS, we might expect a single-channel current of 10 pA at +50 mV (Figure 4). According to this, the whole-cell current demonstrated herein is very low, indicating a very small open probability of the BK_Ca_ channel. The results are also consistent with the BK_Ca_-channel activity at +50 mV shown in Figure 5. This remark applied to the RFM-stimulated current, which was just twice the control. Therefore, it is worthy of being further investigated, whether RFM-stimulated *I*_K(Ca)_ magnitude could be greatly enhanced as the channel number in different cell types is raised.

The IC_50_ values for the RFM-mediated inhibition of the peak and late *I*_Na_ in this study were estimated to be 22.7 μM and 3.1 μM, respectively. Our findings showed the effectiveness of RFM in causing the differential inhibition of the peak and late components of the *I*_Na_ in GH_3_ cells and in hippocampal mHippoE-124 neurons (see the Supplementary Information). In the continued presence of Tef, the addition of RFM led to an attenuation of the Tef-activated *I*_Na_. It also needs to be emphasized that BK_Ca_-channel activity has been identified in human cardiac fibroblasts [65]. It is thus possible that the added RFM interacts with BK_Ca_ channels to raise the amplitude of *I*_K(Ca)_ in those cells which might be electrically coupled to heart cells.

Another important finding in this study is the occurrence of the voltage-dependent hysteresis of the activity of single BK_Ca_ channels activated in response to an inverted isosceles-triangular ramp pulse, in situations where the intracellular surfaces of detached membrane patches from GH_3_ cells were exposed to varying concentrations of RFM. With the increase in the RFM concentration, the strength of the hysteresis exhibited by the channels (i.e., the difference in voltage between the forward and backward limbs at 50% channel open probability) was noticeably enhanced. The results suggest that as the RFM concentration was increased, the voltage-dependent change in the channel activity (i.e., the voltage sensor domain) was intrinsically modified so that the difference between the V_1/2_ values measured at the downsloping and upsloping ends of the inverted triangular ramp widened. However, no change in the single-channel conductance of the BK_Ca_ channels activated by this ramp pulse was observed with either the absence or presence of various concentrations of RFM. Therefore, it is likely that the observed change in the voltage-dependent hysteresis in the presence of RFM does not occur in the pore region of the channel, although the channel activity exhibited intrinsic hysteresis.

The maximum plasma concentrations of RFM at dosages of 10 mg/kg/day and 30 mg/kg/day have previously been reported to be 4.01 μg/mL (16.8 μM) and 8.68 μg/mL (36.4 μM), respectively [8]. Similar results have also been obtained in other studies [7,8,66]. As such, both IC_50_ values found in its inhibition of *I*_Na_ and EC_50_ in its stimulation of *I*_K(Ca)_ were noted to fall within clinically achieved concentrations. Hence, the explanations for the RFM-mediated perturbation of ionic currents presented in the current study could have therapeutical and pharmacological relevance.

**Figure 9:**
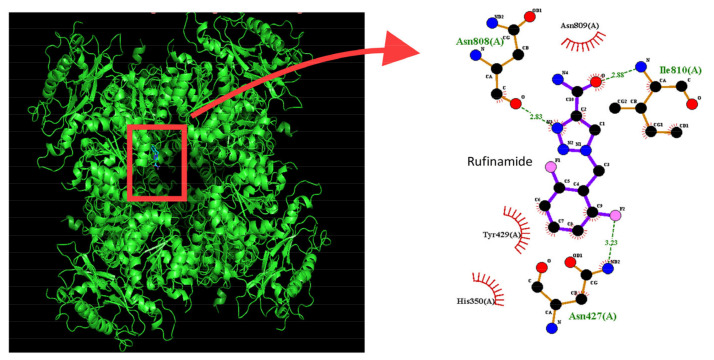
Docking results of K_Ca_1.1 channel and rufinamide (RFM). The protein structure of the K_Ca_1.1 channel was obtained from PDB (PDB ID: 6V3G), while the structure of RFM was acquired from PubChem (Compound CID: 129228). The structure of the K_Ca_1.1 channel was docked with the RFM molecule through PyRx (https://pyrx.sourceforge.io/, URL accessed on 2 November 2022). The diagram of the interaction between the K_Ca_1.1 channel and the RFM molecule was generated by LigPlot^+^ (https://www.ebi.ac.uk/thornton-srv/software/LIGPLOT/, accessed on 2 November 2022). Red arcs with spokes radiating toward the ligand denote the hydrophobic contact, while the green dotted line depicts the hydrogen bond.

In this study, we also conducted a docking study using PyRx software to investigate how RFM and the protein of the K_Ca_1.1 might fit together. The predicted binding sites of the RFM are shown in Figure 9. Given that RFM forms hydrogen bonds with the amino acid residues Asn427, Asn808, and Ile810 and that it forms hydrophobic interactions with the amino acid residues His350, Tyr429, and Asn809, we conclude that RFM can bind to the intracellular domain of K_Ca_1.1 channels and that the RFM-induced binding site is not located in the pore regions of the channels.

## 4. Materials and Methods

### 4.1. Chemicals, Drugs, and Solutions Used in This Work

Rufinamide (RFM, Banzel^®^, Inovelon^®^, R8404, 1-((2,6-difluorophenyl)methyl)-1*H*-1,2,3-triazole-4carboxamide, CAS number: 106308-44-5, C_10_H_8_F_2_N_4_O), E-4031, glibenclamide, tefluthrin (Tef), tetraethylammonium chloride (TEA), and tetrodotoxin (TTX) were acquired from Sigma-Aldrich (Merck, Taipei, Taiwan). Apamin, iberiotoxin, and paxilline were supplied by Alomone (Asia Bioscience, Taipei, Taiwan), and TRAM-34 by Togenesis Technologies (Taipei, Taiwan). For cell preparations, we obtained culture media, fetal bovine/calf serum, horse serum, L-glutamine, and trypsin/EDTA from HyClone^TM^ (Thermo Fisher; Level Biotech, Tainan, Taiwan), and other chemicals, such as CdCl_2_, CsCl, and CsOH, were of analytical research grade.

The ionic composition of the normal Tyrode’s solution used as the extracellular bath solution was (in mM): NaCl 136.5, KCl 5.4, CaCl_2_ 1.8, MgCl_2_ 0.53, glucose 5.5, and HEPES-NaOH buffer 5.5 (pH 7.4). The composition of the intracellular pipette (internal) solution for measuring the ions flowing through the whole-cell K^+^ currents was (in mM): K-aspartate 130, KCl 20, MgCl_2_ 1, Na_2_ATP 3, Na_2_GTP 0.1, EGTA 0.1, and HEPES-KOH buffer 5 (pH 7.2). To measure the *I*_Na_, we substituted K^+^ ions in the internal solution for equimolar Cs^+^ ions, the pH value was adjusted to 7.2 by adding CsOH, and the cells were bathed in Ca^2+^-free Tyrode’s solution containing 10 mM TEA. For single BK_Ca_-channel recordings, the high K^+^ bathing solution was (in mM): KCl 145, MgCl_2_0.53, and HEPES-KOH buffer 5 (pH 7.4), and the pipette solution was (in mM): KCl 145, MgCl_2_ 2, and HEPES-KOH buffer 5 (pH 7.2). A dissociation constant of 0.1 μM for EGTA and Ca^2+^ (at pH 7.2) was the basis for estimating the cytosolic-free Ca^2+^ concentration. The bath and pipette solutions were filtered on the day of use with a syringe filter equipped with a 0.22-μm Supor^®^ nylon membrane (#4612; Pall Corp; Genechain Biotechnology, Kaohsiung, Taiwan).

### 4.2. Cell Preparations

The GH_3_ pituitary cell line was supplied by the Bioresources Collection and Research Center (BCRC-60015; Hsinchu, Taiwan). The GH_3_ cell line was maintained by growing cells in 50 mL plastic culture flasks in 5 mL of Ham’s F-12 medium, supplemented with 2.5% fetal calf serum (*v*/*v*), 15% horse serum (*v*/*v*), and 2 mM L-glutamine. The growth medium was replaced twice a week, and the cells were split into subcultures once a week. Electrophysiological recordings were conducted five or six days after the cells were cultured up to 60–80% confluence.

### 4.3. Electrophysiological Recordings

Shortly before each measurement, GH_3_ cells were gently dispersed, after which, a few drops of cell suspension were quickly placed into a custom-built recording chamber and allowed to settle at the bottom of the chamber. The recording chamber was positioned on the stage of an inverted phase-contrast microscope (Diaphot-200; Nikon; Lin Trading Co., Taipei, Taiwan) which was equipped with a video camera system with a magnification capability of up to 1500× magnification to monitor the cell size during the experiments. The cells were kept in a bath at room temperature (20–25 °C) in normal Tyrode’s solution containing 1.8 mM CaCl_2_. The patch electrodes were prepared from Kimax^®^-51 capillaries with a 1.5–1.8 mm outer diameter (#34500; Kimble; Dogger, New Taipei City, Taiwan) using a two-state PP-830 puller (Narishige; Taiwan Instrument, Tainan, Taiwan), and the tips were then fire-polished with an MF-83 microforge (Narishige). When filled with pipette solution, the resistances ranged between 3 MΩ and 5 MΩ. We performed standard patch-clamp recordings in the cell-attached, inside-out, or whole-cell configurations using an RK-400 amplifier (Bio-Logic, Claix, France) [67]. Junction potentials, which develop at the electrode tip when the composition of the intracellular solution differs from that of the bath, were nulled before the start of the formation of each gigaseal, and the junction potential corrections were then applied to the whole-cell data. During measurement, the recorded signals were stored online at 10 kHz or more with an ASUSPRO-BN401 LG laptop computer (ASUS, Tainan, Taiwan) equipped with a Digidata-1440A device (Molecular Devices; Advanced Biotech, New Taipei City, Taiwan) and controlled with the pCLAMP 10.6 software (Molecular Devices) [68].

### 4.4. Whole-Cell Current Analyses

To determine the percentage increase in the *I*_K(Ca)_ evoked by the presence of RFM, the amplitude of the current at the concentration of 300 μM RFM was taken as 100%, and the current amplitudes during cell exposure to different concentrations of RFM (1–300 μM) were analyzed and compared. To measure the *I*_K(Ca)_, we kept cells bathed in normal Tyrode’s solution containing 1.8 mM CaCl_2_, and a depolarizing voltage command pulse from 0 mV to +50 mV was applied. The amplitudes of the *I*_K(Ca)_ measured during the addition of RFM were compared with those measured after the subsequent addition of paxilline (1 μM). The concentration-response data for the activation of the *I*_K(Ca)_ were then fitted to the modified Hill equation (i.e., a multi-parameter logistic equation) using the least-squares method, as follows:

Percentage increase %=CnH×EmaxCnH+EC50nH

where [*C*] is the RFM concentration added; *E_max_* is the maximal stimulation of the *I*_K(Ca)_ (i.e., the paxilline-sensitive current) caused by the addition of RFM; and *n_H_* and *EC*_50_ are the Hill coefficient and the RFM concentration required to achieve 50% stimulation, respectively.

To determine the concentration-dependent inhibition of the peak and late components of the *I*_Na_ caused by RFM, the GH_3_ cells were kept bathed in Ca^2+^-free Tyrode’s solution, to which 1 μM TTX and 0.5 mM CdCl_2_ were added. The tested cell was voltage-clamped at –80 mV and a 30 ms step depolarization from –80 mV to −10 mV was delivered to it. The amplitudes of the currents (recorded at the beginning and end of the depolarization) evoked by the depolarizing voltage command pulse to –10 mV were measured during the control period (i.e., in the absence of RFM) and during cell exposure to varying concentrations of RFM (0.1–300 μM). The concentration required to suppress 50% (i.e., *IC*_50_) of the peak and late *I*_Na_ was estimated on the basis of the goodness of fit test with another modified form of the Hill equation, as follows:

Relative amplitude=RFM−nH×1−aIC50−nH+RFM−nH+a

where [RFM] represents the different concentrations of RFM used; and *n_H_* and *IC*_50_ are the Hill coefficient inherent to the concentration-response relationship and the concentration at which the inhibition of 50% of the peak or late *I*_Na_ is observed, respectively. At this point, the maximal inhibition (1−*a*) of the peak and late *I*_Na_ was also estimated.

The *I–V* relationship of the peak *I*_Na_ obtained with and without the addition of RFM was derived and fitted to a Boltzmann function given as:

$$\frac{I}{I_{max}}=\frac{G}{1+exp\left[-\left(V-V_{h}\right)/k\right]}\times \left(V-E_{rev}\right)$$

where *V* is the voltage in mV; *E_rev_* is the reversal potential of the *I*_Na_ (fixed at +45 mV); *G* is the Na^+^ conductance expressed in nS; *I* is the current expressed in pA; and *V_h_* and *k* are the gating parameters.

### 4.5. Single-Channel Analyses

The amplitudes of single BK_Ca_ channels identified in the GH_3_ cells were determined by fitting Gaussian distributions to the amplitude histograms of the closed (resting) and open states. The open-state probability of a channel in a patch was expressed as N·*P_O_* and was estimated using the following equation:

N·PO=A1+2A2+…+nAnA0+A1+A2+…+An

where *A*_0_ indicates the area under the curve of an all-points histogram that corresponds to the closed (resting) state; *A*_1_ … *A_n_* are the histogram areas indicating the levels of a distinct open state for 1 to *n* channels in the patch; and N represents the number of active channels in the patch. To perform the analysis of the open and closed time in the channel, only one single channel in the patch was used.

The relationship between the membrane potential and the relative probability of the BK_Ca_ channels being in the open state in response to a triangular ramp pulse with and without the addition of RFM was established and well fitted to the Boltzmann equation (or the Fermi-Dirac distribution) as follows:

relative N·PO=n1+exp−(V−V12)qFRT

where N is the number of channels in the patch; *n* is the maximum relative N·*P_O_*; *V* is the membrane potential expressed in mV; *V*_1/2_ and *q* are the potential for half-maximal activation and the apparent gating charge, respectively; and *F*, *R*, and *T* are the Faraday constant, the universal gas constant, and the absolute temperature, respectively.

To evaluate the effect of RFM on the strength of the hysteresis exhibited by the BK_Ca_ channels, a 1.6 s inverted triangular ramp pulse from +180 to −180 mV with a ramp speed of ±450 mV/s at a rate of 0.05 Hz was applied to the detached patch of membrane by converting the electrical signal from digital to analog. To establish the relative probability of the channels opening in response to the exposure of the cell to different concentrations of RFM, the amplitudes of the current in each single channel evoked by 20-voltage ramps were averaged, and each point of the averaged current was divided by the single-channel amplitude measured for each potential after a correction was performed for a leak component. The number of active channels in the patch, N, was counted at the end of the experiments by introducing a solution with 100 μM Ca^2+^, which was then used to normalize the open-state probability. To acquire values for the gating charge and the half-maximal activation of the current, the curve obtained at the descending (forward), or ascending (backward) limb of the inverted triangular ramp pulse was approximately fitted with a Boltzmann function as described above.

### 4.6. Curve-Fitting Procedures and Statistical Analyses

The curves were fitted to the experimental data by performing a linear or nonlinear regression (i.e., exponential or sigmoid function) using the pCLAMP 10.7 software (Molecular Devices), the 64-bit OriginPro 2021 software (OriginLab^®^; Scientific Formosa, Kaohsiung, Taiwan), or the Microsoft Excel 2013 software. The results of the analyses of the data, which consisted of different types of ionic currents, are presented as the mean ± standard error of the mean (SEM). The sample size (n) indicates the number of cells examined. SEM error bars were plotted. The differences are considered statistically significant when *p* < 0.05, as indicated in the legends for the figures.

## 5. Conclusions

The important findings of this study are: (a) the presence of RFM caused an increase in the amplitude of the *I*_K(Ca)_, the size of which depended on the RMF concentration; (b) the RFM-activated *I*_K(Ca)_ was reversed with the addition of iberiotoxin and paxilline but not apamin or glibenclamide; (c) RFM added to the cytosolic surface of the detached membrane patch increased the open-state probability of BK_Ca_ channels, but no evident change in single-channel conductance was detected in its presence; (d) the RFM-induced increase in the BK_Ca_-channel activity was suppressed by paxilline, but not by TRAM-34; (e) the presence of RFM enhanced the strength of the hysteresis exhibited by the BK_Ca_ channels activated by an inverted isosceles-triangular ramp pulse; and (f) the presence of RFM differentially suppressed the peak and late components of the *I*_Na_ activated by the rapid depolarization of the cell membrane. Taken together, these findings suggest that the RFM-mediated stimulation of BK_Ca_ channels demonstrated herein brings to light an as-yet unidentified but important ionic mechanism underlying the actions produced by RFM, through which it affects neuronal activities, including neuronal excitability.

## Data Availability

Data are available upon request from the corresponding authors.

## Supplementary Materials

The following supporting information can be downloaded at: https://www.mdpi.com/article/10.3390/ijms232213677/s1.
