# Supplementary material for: Rufinamide, a Triazole-Derived Antiepileptic Drug, Stimulates Ca2+-Activated K+ Currents While Inhibiting Voltage-Gated Na+ Currents
Source: Int J Mol Sci. 2022 Nov 8;23(22):13677. doi: 10.3390/ijms232213677 (PMC9697614; doi:10.3390/ijms232213677)
Supplement: Supplementary file 1 [file ijms-23-13677-s001.zip › ijms-1971144-supplementary.pdf]

Supplementary Information

Effect of rufinamide (RFM) on voltage-gated Na^+ current (I_{Na}) in mouse hippocampal (mHippoE-14) neurons

In another set of experiments, we sought to determine if the presence of RFM can result in any perturbations on peak and late components of I_{Na} in hippocampal mHippoE-14 cells. The preparation of hippocampal neurons is illustrated in **Materials and Methods**. As cells were depolarized from -100 to -10 mV with a duration of 30 msec, the peak and late amplitude of I_{Na} was robustly evoked with a rapid activating and inactivating time course. One minute of exposing cells to 3 or 10 μM RFM progressively and differentially decreased the peak and late I_{Na} (**Supplementary Figure S1**). For example, the presence of 10 μM RFM sigificantly decreased the peak and late I_{Na} to 1234±89 and 21±6 pA (n = 8, $P < 0.05$) from control values of 1623±102 and 49±9 pA (n = 8), respectively. After washout of RFM, peak and late amplitudes of I_{Na} were returned to 1611±98 and 48±9 pA (n = 8). It is clear from the present observations that similar to the results found in pituitary GH_3 cells, the presence of RFM is effective in suppressing peak and late I_{Na} in mHippoE-14 neurons and that RFM decreased late I_{Na} to a greater extent than peak I_{Na}.

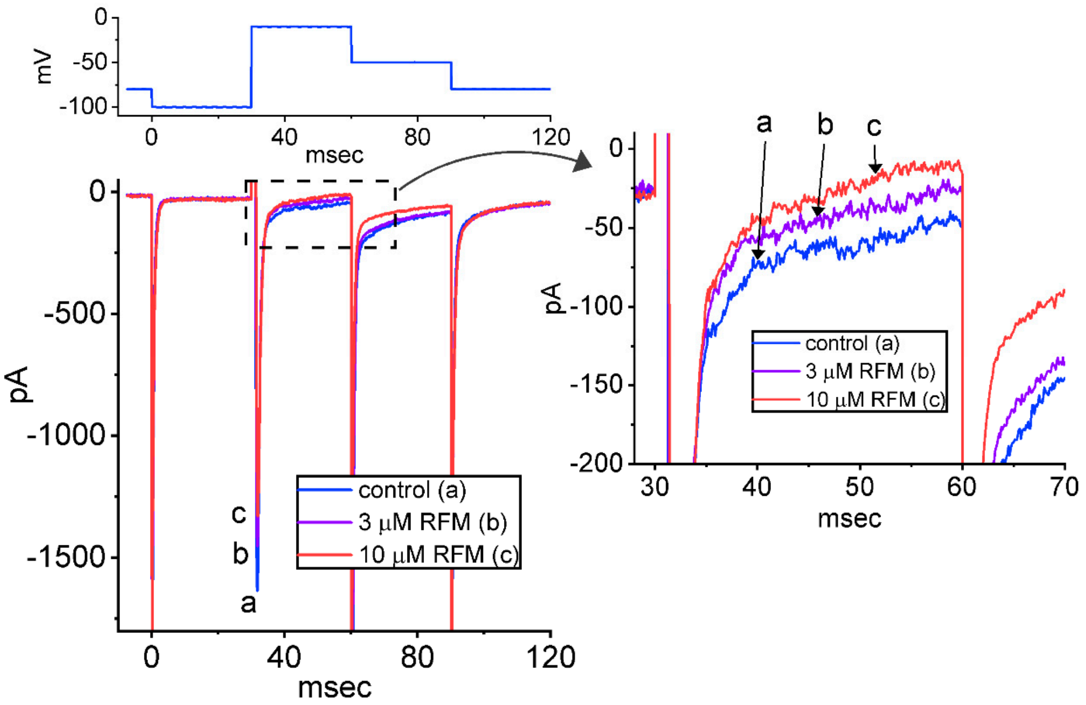

Supplementary Figure S1. Inhibitory effect of RFM on I_{Na} in hippocampal mHippoE-14 cells. In these experiments, cells were immersed in Ca^{2+}-free, Tyrode's solution containing 10 mM tetraethylammonium chloride and 0.5 mM $CdCl_2$, and the recording electrode was filled with Cs^+-containing solution. The upper part shows the voltage-clamp protocol applied. Current trace labeled a is control, and those labeled b and c were acquired in the presence of 3 and 10 μM RFM, respectively. Current traces at a faster time scale in the right side indicate an expanded record from dashed box in the left side.
